# On the Role of Protein Disulfide Isomerase in the Retrograde Cell Transport of Secreted Phospholipases A_2_

**DOI:** 10.1371/journal.pone.0120692

**Published:** 2015-03-12

**Authors:** Jernej Oberčkal, Lidija Kovačič, Jernej Šribar, Adrijana Leonardi, Klemen Dolinar, Anja Pucer Janež, Igor Križaj

**Affiliations:** 1 Department of Molecular and Biomedical Sciences, Jožef Stefan Institute, Ljubljana, Slovenia; 2 Department of Chemistry and Biochemistry, Faculty of Chemistry and Chemical Technology, University of Ljubljana, Ljubljana, Slovenia; Universidad de Costa Rica, COSTA RICA

## Abstract

Following the finding that ammodytoxin (Atx), a neurotoxic secreted phospholipase A_2_ (sPLA_2_) in snake venom, binds specifically to protein disulfide isomerase (PDI) *in vitro* we show that these proteins also interact in living rat PC12 cells that are able to internalize this group IIA (GIIA) sPLA_2_. Atx and PDI co-localize in both differentiated and non-differentiated PC12 cells, as shown by fluorescence microscopy. Based on a model of the complex between Atx and yeast PDI (yPDI), a three-dimensional model of the complex between Atx and human PDI (hPDI) was constructed. The Atx binding site on hPDI is situated between domains b and b’. Atx interacts hPDI with an extensive area on its interfacial binding surface. The mammalian GIB, GIIA, GV and GX sPLA_2_s have the same fold as Atx. The first three sPLA_2_s have been detected intracellularly but not the last one. The models of their complexes with hPDI were constructed by replacement of Atx with the respective mammalian sPLA_2_ in the Atx—hPDI complex and molecular docking of the structures. According to the generated models, mammalian GIB, GIIA and GV sPLA_2_s form complexes with hPDI very similar to that with Atx. The contact area between GX sPLA_2_ and hPDI is however different from that of the other sPLA_2_s. Heterologous competition of Atx binding to hPDI with GV and GX sPLA_2_s confirmed the model-based expectation that GV sPLA_2_ was a more effective inhibitor than GX sPLA_2_, thus validating our model. The results suggest a role of hPDI in the (patho)physiology of some snake venom and mammalian sPLA_2_s by assisting the retrograde transport of these molecules from the cell surface. The sPLA_2_–hPDI model constitutes a valuable tool to facilitate further insights into this process and into the (patho)physiology of sPLA_2_s in relation to their action intracellularly.

## Introduction

Secreted phospholipases A_2_ (sPLA_2_s, EC 3.1.1.4) form an assemblage of enzymes secreted by cells that hydrolyse glycerophospholipids to *sn*-2 lysoglycerophospholipids and fatty acids [1]. The fundamental role of these molecules in physiology is reflected in the fact that they have been described from viruses to mammals. Based on their structural characteristics they are classified into eleven groups [2]. In humans there are ten enzymatically active sPLA_2_s known, GIB, GIIA, GIIC-F, GIII, GV, GX and GXIIA, and two sPLA_2_s apparently without enzymatic activity, GXIIB and otoconin-90/95. Expression patterns of these molecules in mammalian tissues differ, so their functions appear to be non-redundant [3]. Either by enzymatic activity or by binding to soluble or membrane receptors [4] these molecules are implicated in many physiological and pathological settings, such as innate immunity, asthma, atherosclerosis, inflammatory diseases, Alzheimer’s disease, neuritogenesis, neurotransmitter release and various forms of cancer [5–8]. Despite intensive research on sPLA_2_ pathophysiology, the mechanisms of action of most of the known sPLA_2_-associated phenomena are not well understood. It is still widely supposed that sPLA_2_s act exclusively extracellularly. However, growing evidence concerning the localization of some types of sPLA_2_s inside various cells suggests that they also induce certain cellular processes from inside cells. In compartments other than those leading imported proteins to degradation or *de novo* synthesized proteins to secretion, three mammalian sPLA_2_s have been detected, GIB, GIIA and GV sPLA_2_. GIB sPLA_2_ was found in the nucleus of UIII cells, a stromal cell line derived from normal rat uterus, GIIA in the mitochondria and GV sPLA_2_ in the nucleus and cytoplasm of U251 astrocytoma, PC12 and P388D1 macrophage-like cells [9–12].

Snake venoms are a rich source of GI and GII sPLA_2_s. These are closely related structurally to mammalian sPLA_2_s and thus very useful for studying the function of the latter. Ammodytoxin (Atx), the GIIA sPLA_2_ from the nose-horned viper (*Vipera a*. *ammodytes*) is among the most thoroughly studied snake venom sPLA_2_s. This molecule is neurotoxic, acting at the presynaptic side of the neuro-muscular synapse [13]. It is internalized into the cytosol, synaptic vesicles and mitochondria of the motoneuron-like NSC34 cells [14], and into the mammalian motor nerve terminal, where it becomes associated with mitochondria and cytoplasmic vesicles [15]. Specific pathways must exist by which Atx crosses the plasma membrane to enter the cytosol and mitochondria of nerve cells and which are likely to share at least some common characteristics with pathways that mammalian orthologues are using. It is important to answer the question as to how Atx crosses cellular membranes, not only in describing the molecular mechanism of its neurotoxicity but also in shedding more light on the pathophysiological function of some homologous mammalian sPLA_2_s.

Several years ago we demonstrated that Atx binds strongly *in vitro* to protein disulfide isomerase (PDI) [16]. PDI is an oxido-reductase, located in the lumen of the endoplasmic reticulum (ER) [17]. We proposed then that it could be implicated in the retrograde trafficking of this toxin in a similar way as revealed in the case of some other protein toxins [18–22]. Besides assisting Atx to move retrogradely from Golgi apparatus to ER, PDI can also help Atx to translocate across the ER membrane. Transport between Golgi apparatus and ER is mediated by cycling of the KDEL receptor. KDEL, or a similar sequence, is located specifically at the C-termini of the ER-resident proteins and prevents them from escaping from the ER lumen. While cholera toxin possesses this signal sequence intrinsically, in its A-subunit, Atx does not but may take advantage of the signal sequence of PDI if complexed with PDI *in vivo*, in the lumen of the ER. The results reported here strongly support the hypothesis that PDI partakes at the retrograde cellular transport of Atx and the related mammalian sPLA_2_s. The sPLA_2_–hPDI model that we present provides a structural insight into the interaction between these proteins thus enabling a targeted study of the sPLA_2_ cell internalization process.

## Materials and Methods

### Materials

Two isoforms of Atx, AtxA and AtxC, having very similar biological activity [13], were used in our experiments. They were purified from *Vaa* venom as described previously [23]. Sulfo-SBED reagent (sulfosuccinimidyl-2-[6-(biotinamido)-2-(*p*-azidobenzamido)-hexanoamido] ethyl-1,3′-dithio propionate), streptavidin conjugated with horseradish peroxidase (SA—HRP) and monomeric avidin were purchased from Pierce. The fluorogenic substrate PyPG for measuring phospholipase activity was prepared from 1-hexadecanoyl-2-(1-pyrenedecanoyl)-*sn*-glycero-3-phosphoglycerol (Molecular Probes) as described [24]. Bovine α-chymotrypsin, Triton X-100, Tween 20, dithiothreitol (DTT), N-2-hydroxyethylpiperazine-N-2-ethanesulfonic acid (HEPES), glutathione (reduced and oxidized forms) and fatty acid-free bovine serum albumin (BSA) were from Sigma. Human group V and group X sPLA_2_ (hGV and hGX sPLA_2_) were produced as described [25]. Recombinant wild type yeast PDI (wt yPDI) and its domains were a gift from Dr. William J. Lennarz, Stony Brook University, New York, USA. These proteins were produced as described [26]. Recombinant human PDI-A1 (hPDI-A1) was from Biorbyt. Rabbit polyclonal anti-PDI IgGs were form Enzo Life Sciences. Mouse polyclonal anti-calmodulin (CaM) IgGs were from Abcam. Protein-A Sepharose was from Amersham Biosciences. BM Chemiluminescence and Lumi-Light^PLUS^ Western Blotting Substrate were from Roche Diagnostics. Other chemicals were obtained mainly from Sigma and Serva and were of at least analytical grade.

### PC12 cell culture

PC12 is a cell line derived from a pheochromocytoma of the rat adrenal medulla [27]. PC12 cells ATCC CRL-1721 (American Type Culture Collection, USA) were grown at 37°C under 5% (v/v) CO_2_ in 10-cm culture plates in F12K (Kaighn's modification of Ham's F-12) growth medium (Gibco, USA) containing 15% (v/v) horse serum, 2.5% (v/v) fetal bovine serum, 100 units/mL of penicillin and 100 μg/mL streptomycin (culture medium). To induce differentiation, nerve growth factor (NGF) was added to the growth medium (100 ng/mL). After 48 hours, the cells were washed with phosphate buffered saline (PBS), fresh NGF-containing growth medium was added, and the cells grown for a further 48 hours. For microscopy, cells were plated on poly-L-Lys coated coverslips and, for viability and mitochondrial membrane potential assays, the cells were plated on 96-well plates.

### Testing the influence of Atx on PC12 cells

The influence of Atx on the morphological integrity of non-differentiated (ND) and NGF-differentiated (NGFD) cells was examined. These cells were grown for 120 min at 37°C in the presence of 100 nM AtxC, then inspected for their morphological integrity under a phase contrast microscope Axio Observer Z1 (Carl Zeiss, Germany) using a Plan-Apochromat 40x/0.95 Corr Ph3 M27 objective.

The effect of Atx on the mitochondrial potential of PC12 cells was evaluated. The cells (ND and NGFD), in 96-well plates (10^4^ cells per well), were incubated in the dark with 2 μM JC-1 dye (Life Technologies, USA) in F12K growth medium for 20 min at 37°C under 5% (v/v) CO_2_. After washing with the medium, the cells were incubated in the dark for a further 30 min at 37°C under 5% (v/v) CO_2_ in the growth medium alone (control) or in the medium supplemented with either 100 nM AtxA or 50 μM CCCP (mitochondrial uncoupler carbonyl cyanide m-chlorophenyl hydrazone). The green fluorescence of JC-1 at 529 nm and the red fluorescence at 590 nm of aggregated JC-1 were measured in triplicate samples on a SAFIRE microplate reader (Tecan, Austria), using the excitation wavelength 488 nm. The mitochondrial membrane potential was exhibited as the ratio of red to green signals of the cells in the presence of the substance investigated relative to the same ratio in the absence of this substance.

The influence of Atx on the metabolic activity of PC12 cells was measured as follows. PC12 cells (ND and NGFD) in 96-well plates (5×10^4^ cells per well) were incubated without (control) or with either 100 nM AtxA or 1% (m/v) Triton X-100 in culture medium at 37°C and 5% (v/v) CO_2_. After different incubation times, the cells were washed twice with F12K growth medium and incubated in the dark for a further 2 hours at 37°C under 5% (v/v) CO_2_ with MTS (3-(4,5-dimethylthiazol-2-yl)-5-(3-carboxymethoxyphenyl)-2-(4-sulfophenyl)-2H-tetrazolium) (20 μL per 100 μL F12K growth medium) prepared according to the manufacturer's (Promega, USA) instructions. After 2 hours, the absorbance at 490 nm was measured on a SAFIRE microplate reader. The background absorbance was measured on wells containing MTS in F12K growth medium without cells. Viability of cells was calculated from A_490_ corrected for background absorbance and presented relative to the control cells. Measurements were performed in triplicate.

### Testing internalization of Atx into PC12 cells

The internalization test used by Jenko Pražnikar et al. [14] was used. In brief, sulfo-SBED-Atx, synthesized as described [28,29], was incubated in the dark with ND and NGFD PC12 cells in culture. Following photo-activation of the Atx probe, cellular proteins in tight complexes with the probe were labelled with biotin. Cells were lysed, the biotinylated proteins selectively extracted using avidin beads and probed for anti-CaM IgG cross-reactivity by Western blotting.

### Labelling PDI in PC12 cells with sulfo-SBED-Atx

Intact PC12 cells (both ND and NGFD), plated on 10 cm plates at 80–90% confluence, were treated with sulfo-SBED-Atx at a final concentration of 100 nM for 1, 2, 5, 15, 30 and 60 min in Hank's balanced salt solution supplemented with 1.26 mM Ca^2+^ (Ca^2+^/HBSS) in the dark at 37°C and 5% (v/v) CO_2_. Subsequently, cells were cooled on ice, exposed for 5 min to five 15 W 312 nm UV lamps from a distance of 5 cm, washed twice with Ca^2+^/HBSS, 1.5% (w/v). Triton X-100/Ca^2+^/HBSS extracts were then prepared. Experiments were performed in duplicate using cells of the same passage. The extracts were centrifuged for 45 min at 14.000 × *g* and 4°C. Supernatants were incubated with 375 μL monomeric avidin beads for 1 hour at 4°C. The beads were thoroughly washed with Ca^2+^/HBSS containing 0.1% (w/v) Triton X-100 and biotin containing proteins then eluted with 2 mM *D*-biotin. The fractions were concentrated and the proteins recovered as described [30]. The resulting pellets were dissolved in SDS-PAGE loading buffer and analyzed as described below. To standardize the amount of samples applied the intensity of the band corresponding to a protein of 85 kDa (present in control and sulfo-SBED-Atx labelled samples analysed on SDS-PAGE with silver staining) was used as a measure. The position of biotinylated PDI on the gel was determined immunologically using 0.3 μg/mL rabbit polyclonal anti-PDI antibodies.

### Immunofluorescence studies

AtxA was dissolved in DMSO (2 mg/mL final concentration) and Alexa Fluor 546 (Molecular Probes Alexa Fluor546 Protein Labelling Kit, Life Technologies, USA) was added to a final protein: dye molar ratio of 1: 1.5 in the presence of 100 mM triethylamine. After 1 h shaking in the dark at room temperature, taurine was added (5 mM final concentration) and the mixture incubated for a further 30 min. The reaction products were separated on RP-HPLC using an Aquapore BU 300 column (30 mm × 4.6 mm) (PerkinElmer, USA) equilibrated with 5% (v/v) solvent B (90% (v/v) acetonitrile and 0.1% (v/v) trifluoroacetic acid (TFA) in water) in solvent A (0.1% (v/v) TFA in water). After application of the sample, the column was washed with 5 mL of solvent A at a flow rate of 1 mL/min and the bound proteins then eluted with a gradient of solvent B in solvent A from 5% to 20% for 3 min, then from 20% to 60% B for 20 min and finally to 100% B for 3 min. Their absorbance was monitored at 215 nm. As selected by mass spectrometry analysis, the Alexa mono-derivative of AtxA (^546^Alexa-Atx) was further characterized to confirm that its enzymatic activity and ability to bind to CaM do not differ from those of the native toxin.

PC12 cells on poly-L-lysine coated coverslips were incubated at 37°C and 5% (v/v) CO_2_ in the presence of 100 nM ^546^Alexa-Atx for 1, 2, 5, 15, 30, and 60 min. The cells were first washed three times with Dulbecco's phosphate buffered saline (DPBS) at room temperature and then fixed for 10 min in 4% (w/v) paraformaldehyde in DPBS at room temperature. After fixing, the cells were washed for 5 min with ice-cold DPBS, permeabilized for 5 min with ice-cold 0.1% (w/v) Triton X-100 in DPBS and then incubated at room temperature in blocking solution, containing 0.5% (w/v) fish skin gelatine and 10% (w/v) fetal bovine serum in DPBS. Coverslips were then immersed into primary anti-PDI antibody solution (diluted 1: 300 in blocking solution). After 60 min incubation at room temperature, the cells were rinsed three times for 5 min each in DPBS and then incubated for 60 min at room temperature in secondary antibody solution (Alexa Fluor 488 Goat Anti-Rabbit, diluted 1: 2.000 in blocking solution) and, finally, washed three times 5 min each in DPBS. Excess fluid was removed and the slides mounted using ProLong Gold antifade reagent and left to cure. After 24 hours at room temperature in the dark, the edges of the coverslips were sealed with nail polish and analyzed on an inverted confocal laser scanning microscope (Axio Observer Z1 LSM 710, Carl Zeiss, Germany) with a Plan-Apochromat 63/1.40 oil objective. Fluorophores were excited sequentially using Ar (488 nm) and He-Ne (543 nm) lasers. The emitted light was collected through SP 545 and LP 545 filters. Stacks of fluorescent images were acquired and co-localization of the red and green signal calculated with ImageJ [31] and co-localization plugin JACoP [32]. The signal threshold was determined from autofluorescence of the control cells, and was set at 20% of the maximum signal for both red and green channels. Co-localization was represented by Manders' coefficient, *i*.*e*. the ratio of the summed intensities of pixels from the green channel, for which the intensity in the red channel is above zero, to the total intensity in the green channel.

### Preparation of sulfo-SBED-Atx—yPDI conjugates and their analysis

Equimolar amounts of sulfo-SBED-Atx and wt yPDI or its domains in 75 mM HEPES/HCl, pH 8.2, 150 mM NaCl and 2 mM CaCl_2_ (labelling buffer) were mixed to give 7 μM concentration of each in a final volume of 50 μL. In competition binding assays, 0.5 μM yPDI or 0.2 μM wt hPDI was incubated with sulfo-SBED-Atx (0.5 μM) in the dark, in the absence or the presence of the following competitors: AtxC (50 μM), GV sPLA_2_ (18 μM) or GX sPLA_2_ (13 μM). For mapping the Atx—yPDI interaction surface, the final volume of the reaction mixture was 1 mL. After 60 min incubation at room temperature in the dark, the mixtures were irradiated for 10 min on ice by five 15 W UV lamps at 312 nm from a distance of 5 cm.

Sulfo-SBED-Atx labelled samples were analyzed on SDS-PAGE under reducing conditions. Proteins were transferred from the gel to the PVDF or nitrocellulose membrane by Western blotting in a tank filled with Towbin buffer (25 mM Tris/HCl, 192 mM glycine, 0.1% (w/v) SDS and 20% (v/v) MeOH) for 90 min at 200 mA. On the membrane the biotinylated proteins were detected by SA—HRP and Lumi—Light^PLUS^ Western Blotting Substrate or the BM Chemiluminescence Western blotting detection system according to the manufacturer’s instructions. Kodak BioMax Light Film was used for light capturing (Sigma—Aldrich).

### Isolation and identification of biotinylated peptides

The sulfo-SBED-Atx—yPDI conjugate in 1 mL was cleaved at 37°C with three consecutive additions of 1% (w/w) α-chymotrypsin in 30 min intervals. 10% of the digested reaction mixture was analyzed on a reversed-phase high-pressure liquid chromatography (RP-HPLC) system using a Chrompack C18 (100 × 3.0 mm) column equilibrated with 0.1% (v/v) TFA in water (solvent A). After application of the sample, the column was washed with 5 mL of solvent A at a flow rate of 1 mL/min and the bound peptides then eluted with a gradient of 0% to 60% solvent B (90% (v/v) acetonitrile and 0.1% (v/v) TFA in water) in solvent A in 30 min and then to 100% B in 12 min. Absorbance was monitored at 215 nm. To isolate the biotinylated peptides, 90% of the chymotrypsin-digested sulfo-SBED-Atx—yPDI was added to 150 μL monomeric-avidin beads equilibrated with labelling buffer. The beads were then thoroughly washed with the labelling buffer, followed by a short wash with water. The bound biotinylated peptides were stripped off the beads with 0.1% (v/v) TFA in water and separated on RP-HPLC, using the same protocol as described above for the separation of the 10% of the total digestion reaction mixture. 10% of the material not bound to avidin beads was also analyzed under identical conditions. Isolated biotinylated peptides were N-terminally sequenced by automated Edman degradation on an Applied Biosystems Procise 492A sequencing system.

### Modelling the tridimensional structure of the Atx—yPDI complex

All water molecules were removed from the crystal structures of yPDI (PDB ID 2b5e and 3boa [26]) and AtxA (PDB ID 3g8g [33]). Models of the Atx—yPDI complex were generated using two protocols. The rigid-body docking protocol Hex 5.1 [34] (http://www.csd.abdn.ac.uk/hex) was carried out as described [29]. Steric clashes between side-chain atoms were removed by energy minimization (the root mean square gradient for energy conversion was set to 3.0 kcal/mol/Å) with the Tinker Molecular Modeling Package [35] (http://dasher.wustl.edu/tinker/), using CHARMM27 force field parameters [36]. The high ambiguity-driven docking (HADDOCK) protocol has been run as described [37–39]. For HADDOCK trials, the default parameters on the web server version of HADDOCK (http://haddock.science.uu.nl/services/HADDOCK/haddock.php) [39] were used with the setting of passive residues (*i*.*e*. residues not involved in the interaction) identified from the sequences of the surface exposed biotinylated peptides. Both approaches resulted in two models. In the first model Atx binds to the surface of yPDI at a site between its domains b and b’ (bb’-binding site) while, in the second model, it binds between its domains a’ and c (a’c-binding site). In this way, the interaction of several other sPLA_2_s, structurally similar to Atx, with both yPDI and hPDI was modelled. Coordinates of porcine GIB (pGIB) sPLA_2_ (ID 1pir), hGIIA sPLA_2_ (ID 1pod), hGX sPLA_2_ (ID 1le7) and hPDI (ID 4el1), were obtained from the Protein Data Bank, while the model of hGV sPLA_2_ was constructed as described [29]. All water molecules and ions were removed from the three-dimensional (3D) structures. sPLA_2_ structures were superimposed on the structure of Atx in the bb’ and a’c models of the Atx—yPDI complex. In the same way, the hPDI structure was superimposed on the yPDI structure in the bb’-model of the Atx—yPDI complex. In the thus obtained initial structures, potential active residues (*i*.*e*. residues constituting the interaction area or being situated not more than 10 Å from it) were identified. Semi-flexible segments were manually defined (each PDI domain represented one segment) while passive residues were defined automatically. The HADDOCK procedure was then performed with other parameters set to default values. Docking trials of the sPLA_2_–yPDI interaction yielded models that differ only slightly in the relative positions of sPLA_2_s and yPDI. The models with the lowest non-bonded interaction energy (*i*.*e*. energy of interactions between atoms that are more than three bonds apart) and similar interaction sites were chosen.

In the case of hPDI, no structural restraints were available. Choice of the best Atx—hPDI model was therefore made based on the similarity of the interaction site with that of the Atx—yPDI model and on lowest non-bonded energy criteria. Models of other sPLA_2_–hPDI complexes were then evaluated by comparing them to the chosen Atx—hPDI model. The lowest energy sPLA_2_–hPDI models that had interaction sites similar to that of Atx in the hPDI model were chosen. In all cases, excepting that of hGX sPLA_2_, the process of choosing the best sPLA_2_–hPDI model was very straightforward, since the models with the same interaction site as Atx had the lowest non-bonded energy. For hGX sPLA_2_, the respective sPLA_2_ in the majority of models was offset significantly relative to Atx. Models in which hGX sPLA_2_ exhibited the same interaction site as Atx in the Atx—hPDI complex had significantly higher non-bonded energies; we therefore chose the lowest energy model.

### Measurement of phospholipase activity using the fluorogenic substrate PyPG

The phospholipase activity of AtxC, in the presence or the absence of yPDI, was determined as described [40] in 50 mM Tris/HCl, pH 7.4, 50 mM KCl, 1 mM CaCl_2_, 0.6% (w/v) fatty acid-free BSA.

## Results and Discussion

### Atx does not significantly affect the integrity of PC12 cells

**Fig 1:**
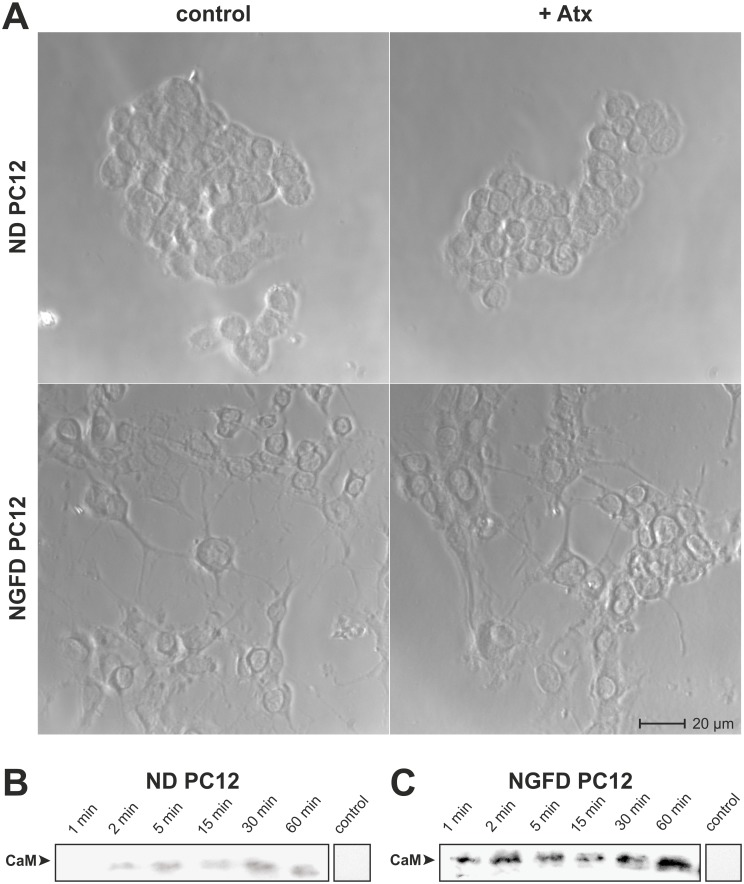
Atx translocates into the cytosol of PC12 cells but does not affect their morphology. (A) Non-differentiated (ND) and NGF-differentiated (NGFD) PC12 cells were incubated with 100 nM AtxC for 120 min at 37°C and then inspected for their morphology under the phase contrast microscope. In control experiments cells were incubated under identical conditions only in the absence of Atx. (B) ND and (C) NGFD PC12 cells were incubated for indicated periods of time with 100 nM sulfo-SBED-Atx in the dark before they were exposed to UV light to trigger photo-cross-linking of the biotin-carrying probe with proteins in close contact. Cells were extracted and biotinylated proteins isolated from extracts on avidin-beads. After reductive SDS-PAGE of biotinylated proteins, these were Western blotted from the gel to a PVDF membrane. The presence of CaM on the PVDF membrane was examined using anti-CaM antibodies and chemiluminescence detection system. Control experiments were performed in an identical way, only without sulfo-SBED-Atx during the 5 min incubation. Experiments were performed in duplicate. Experimental details are given in Materials and Methods.

Influence of Atx on ND and NGFD PC12 cells was evaluated by measuring its effect on the mitochondrial potential, the metabolic activity and the morphology of the cells. While the mitochondrial potential of the cells was about 40% lower in the presence of 100 nM Atx than in its absence, the MTS test showed increased viability of PC12 cells in the presence of Atx (S1 Fig.). These results are in line with previous findings [41]. As part of the complex influence of sPLA_2_s on the cells, they can induce opposing effects, proliferation and apoptosis, in mammalian cells. The morphology of both ND and NGFD PC12 cells was examined, under phase contrast microscopy, following their incubation in the absence and presence of the neurotoxic sPLA_2_. No visual changes were observed in either type of PC12 cells in the presence of 100 nM Atx, as compared with control cells grown in the absence of Atx (Fig. 1A). Under these experimental conditions therefore neither proliferation of the cells by Atx nor the negative effect of the sPLA_2_ on their mitochondria was dominant enough to be reflected in the modified morphology of the cells.

### Atx enters the cytosol of PC12 cells

PC12 cells exhibit properties similar to those of neurons, so they were examined as to whether they constitute an appropriate system for studying the mechanism of internalization of the neurotoxic sPLA_2_. A photo-reactive derivative of Atx, sulfo-SBED-Atx, was employed. As was shown previously, this molecule is able to cross the plasma membrane and enter the cytosol of NSC-34 motoneuron cells, but not that of the non-neuronal HEK-293 [14] and Caco-2 cells (unpublished results). Using the same protocol, our results clearly show that sulfo-SBED-Atx is capable of entering very quickly the cytosol of both ND and NGFD PC12 cells when added to the culture medium. Thus, after about 1 min of incubation with NGFD PC12 cells and about 2 min with the non-differentiated cells, the photo-reactive probe, tagged its cytosolic high-affinity binding protein CaM with biotin (Figs. 1B and 1C). The degree of labelling of CaM by sulfo-SBED-Atx increased with time of incubation. Similarly rapid internalization of Atx in nerve cells has also been observed with rat hippocampal neurons [42] and with motoneuron-like NSC-34 cells [14]. The fact that Atx is able to enter the cytosol of PC12 cells, apparently without affecting them adversely, qualifies them as suitable for studying the pathway of the neurotoxic sPLA_2_ from outside the plasma membrane into the cytosol.

### Atx and PDI are significantly co-localized in PC12 cells

**Fig 2:**
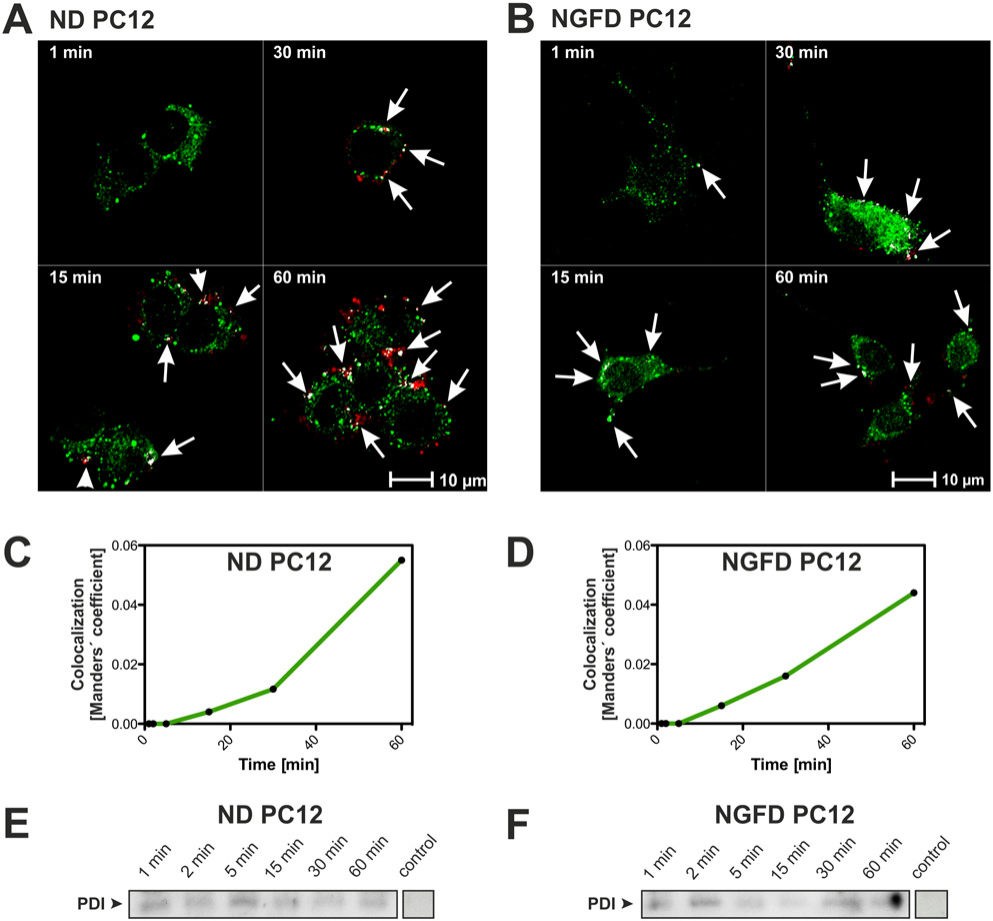
Atx and PDI co-localize and form a complex in living PC12 cells. (A) Non-differentiated (ND) and (B) NGF-differentiated (NGFD) PC12 cells were incubated in the presence of 100 nM ^546^Alexa-Atx (red signal) for indicated times. Cells were fixed and PDI stained with anti-PDI antibodies (green signal). Co-localized green and red pixels are shown in white. Arrows point at most extensive areas of co-localization. Co-localization of Atx and PDI is expressed in terms of Manders' coefficient in time for ND (C) and NGFD (D) PC12 cells. (E) ND and (F) NGFD PC12 cells were incubated with biotin-containing sulfo-SBED-Atx photo-probe for the indicated time, when this was activated by UV light illumination. The photo-probe covalently links to proteins in close contact, thus biotinylating them. Biotinylated proteins were purified from cell extracts using avidin-affinity chromatography. After reductive SDS-PAGE of biotinylated proteins, these were Western blotted from the gel to a nitrocellulose membrane. The presence of PDI on the membrane was examined using anti-PDI antibodies and chemiluminescence detection system. Control experiments were performed in an identical way, only without sulfo-SBED-Atx during the 5 min incubation. All experiments were performed in duplicate. Experimental details are described in Materials and Methods.

The affinity between Atx and PDI has been demonstrated *in vitro* [16]. PDI is a protein in the lumen of the endoplasmic reticulum (ER) known to assist cell invasion by some bacterial toxins such as cholera toxin [18–20]. By analogy, PDI has been proposed as being able to detain and concentrate sPLA_2_ molecules in the ER of certain types of cells and to assist them to translocate across the ER membrane into the cytosol [16]. To examine this suggested role of PDI in the (patho)physiology of Atx we first compared the subcellular localization of Atx and PDI in PC12 cells. ND and NGFD cells were incubated in the presence of ^546^Alexa-Atx (red) for different periods of time, fixed and labelled with anti-PDI antibodies (green) (Figs. 2A and 2B).The distribution of both the red and green signals in the cells was analyzed using confocal laser microscopy, acquiring several optical sections for each sample. Significant red ^546^Alexa-Atx signal was detected after incubation times of 5 min and longer. It was located on the plasma membrane as well as inside the cells, but absent from the nuclei, at least after up to 120 min of incubation. In all cells, the signal appears discrete. This suggests the localization of Atx in vesicular structures, possibly endosomes. In differentiated PC12 cells, ^546^Alexa-Atx was distributed in soma and neurites without evident preference, as reported also for the distribution of Atx and β-bungarotoxins, an sPLA_2_ neurotoxin from the snake *Bungarus multicinctus*, in rat hippocampal neurons [42,43]. Stacks of images of each sample were quantified and the degree of co-localization of the red and the green signal calculated using ImageJ with co-localization plugin JACoP (Figs. 2C and 2D). To eliminate autofluorescence signals, control experiments without the addition of either ^546^Alexa-Atx or ^488^Alexa-conjugated secondary antibodies were used to determine the threshold signal for each channel using Zeiss ZEN Software. The results are represented as the ratio of the number of green pixels that co-localize with red pixels to the total number of green pixels above the threshold. While low co-localization of signals for ^546^Alexa-Atx and PDI was observed at incubation times up to 5 min, co-localization became significant after 30 min. Based on these results we can conclude that PDI and the neurotoxic sPLA_2_ on its way from the external space into the cytosol of the PC12 cell locate regularly at the same sites of the cell.

### Atx encounters PDI in living PC12 cells

To further substantiate the suggested role of PDI in the (patho)physiology of some sPLA_2_s we investigated the possible formation of the complex between PDI and Atx in living cells. Photo-reactive sulfo-SBED-Atx was added to cultures of ND and NGFD PC12 cells and photo-crosslinking initiated at the incubation times indicated in Figs. 2E and 2F. Cell proteins that were in close contact with Atx at these times remained biotinylated. The cells were then lysed, proteins extracted and loaded to an avidin-affinity column that specifically retained only the biotinylated ones. The avidin-binding proteins were then resolved on SDS-PAGE, electroblotted onto the PVDF membrane and inspected for the presence of PDI using specific anti-PDI IgGs. As evident from Figs. 2E and 2F, the photoreactive derivative of Atx encountered PDI in both types of PC12 cells. 1 min after the addition of the sPLA_2_ to the cell culture medium it reached the lumen of the ER of the PC12 cells, bound to and reacted with PDI, in agreement with the rapid downstream labelling of the cytosolic CaM (see above). Interestingly, unlike in the case of labelling CaM by sulfo-SBED-Atx (Figs. 1B and 1C), the extent of biotin tagged-PDI did not increase with time. This could indicate that the interaction between Atx and PDI is transient and that the equilibrium between Atx coming into the lumen of ER and Atx leaving it through the ER membrane into the cytosol is rapidly established. In favour of transient nature of the interaction between Atx and PDI is the apparent dissociation constant of the interaction of 1.27 ± 0.05 μM as determined by surface plasmon resonance [16].

The apparent discrepancy between the results of labelling PDI with sulfo-SBED-Atx, where the product signal remained constant with time, and the result obtained in the co-localization study, where the extent of co-localization increased with time (Figs. 2C and 2D) can be explained by the different nature of the two experiments. In the affinity-labelling experiment only tightly associated molecules of Atx and PDI are observed while, in the co-localization experiment, molecules that are more distant from each other are also indicated. In addition, the methods use differently labelled Atx probes that may have different PDI-binding characteristics. The results, however, strongly support the involvement of PDI in the retrograde cell transport of Atx. A similar function to that of PDI, assistance at moving of sPLA_2_ retrogradely from Golgi apparatus to ER, may be also envisaged in the case of two other ER-proteins, TCBP-49 and crocalbin, which bind, respectively, taipoxin and crotoxin, two snake venom neurotoxic sPLA_2_s resembling Atx in their action [44,45]. Thus it is concluded that the retrograde cellular transport of sPLA_2_s may rely on transient complexing between sPLA_2_s and ER-proteins possessing the ER-retaining sequence at their C-termini.

### Structural characterization of the interaction between Atx and PDI

**Fig 3:**
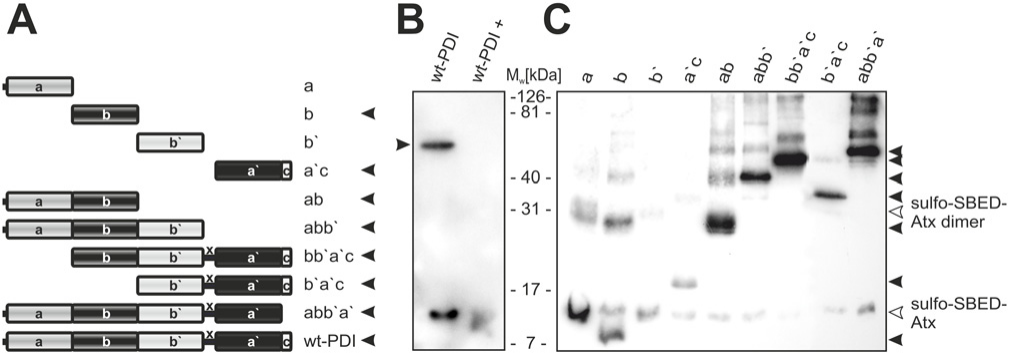
Atx interacts with several domains of yPDI. (A) yPDI is a multi-domain protein. It consists of domains a, a’, b, b’ and c. Domain b’ is linked to domain a’ by a linker sequence x. Separate domains and their fusions, as schematically presented, were employed in affinity-labelling experiments using photo-reactive sulfo-SBED-Atx. Atx photo-probe was incubated with the wt yPDI (wt-PDI) (B) or its various parts (C) for 30 min, followed by the UV light-initiated reaction of the photo-probe with proteins in close contact, which were thus biotinylated. Samples were analysed on SDS-PAGE under reducing conditions, electro-transferred to a PVDF membrane. Biotin-containing bands on the PVDF membrane were detected using streptavidin-HRP. Labelling specificity in (B) was verified with the addition of 100-fold unlabelled Atx over sulfo-SBED-Atx (wt-PDI+). Black arrowheads in (A) point at those elements of yPDI which bind and react with sulfo-SBED-Atx. In (B) and (C) black arrowheads are pointing at biotinylated yPDI-structures. Positions on the membrane with biotinylated monomer and dimer of Atx are pointed at by white arrowheads. Experimental details are described in section Materials and Methods. Experiment was performed in triplicate.

In order to design mutants for further study of the role of PDI in cell trafficking of Atx, the interaction between Atx and PDI was characterized in terms of their structures. yPDI was used for three reasons. (1) As hPDI, it binds specifically to Atx (Fig. 3B). (2) It shares substantial homology to hPDI at the levels of the primary, secondary and ternary structure. For example, the orientation of domains b and b’, important for enzymatic activity and substrate binding, is very similar in both PDIs. The main difference between the two proteins lies in their C-terminal part—the well-structured α-helix in yPDI (domain c) is absent from hPDI [26,46–48]. (3) In addition, separate recombinant domains of this protein and their fusions were available (Fig. 3A).

In order to locate the Atx-interacting site on yPDI, different parts of the protein—individual domains (a, b, a’) and fusions of two or more yPDI domains (a’c, ab, abb’, bb’a’c, b’a’c and abb’a’) (Fig. 3A)—were reacted with the Atx photo-probe. As evident from Fig. 3C, sulfo-SBED-Atx specifically labelled the following yPDI elements: b, ab, a’c and structures containing these elements, but not domains a and b’. This suggests that two binding sites for sPLA_2_ exist on yPDI.

**Fig 4:**
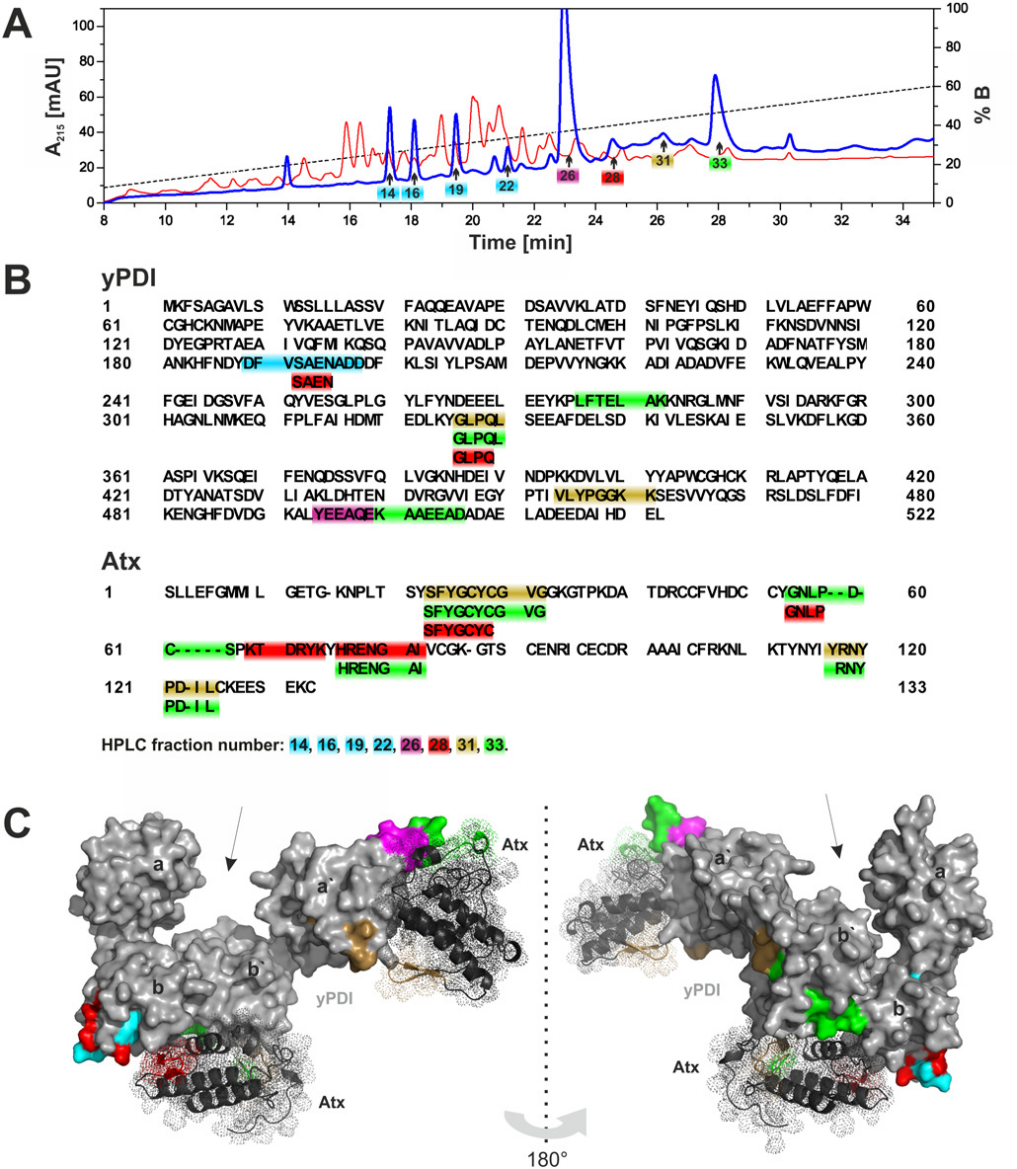
The 3D model of Atx—yPDI complex. (A) Covalent complex of sulfo-SBED-Atx and yPDI was digested with α-chymotrypsin and 10% of the reaction mixture (whole sample) was chromatographed on a Chrompack C18 (100 × 3.0 mm) HPLC column (red line) equilibrated in 0.1% (v/v) TFA and eluted with a gradient of 0.1% (v/v) TFA containing 90% (v/v) acetonitrile. From the rest of the whole sample biotin-containing peptides were isolated on avidin-beads and analysed on HPLC under identical conditions as the whole sample (blue line). Arrows point at fractions containing the major biotinylated peptides. (B) The major biotinylated peptides were identified by N-terminal sequencing. Their positions in primary structures of yPDI or Atx are marked by different colours. A colour designates yPDI and Atx peptides identified in the same fraction. Such peptides are covalently cross-linked and lie close together in the complex, at the rim of the interaction area between yPDI and Atx. (C) Modelling using rigid-body docking protocol Hex 5.1, HADDOCK procedure and experimentally defined structural restraints resulted in two solutions for Atx binding to yPDI. According to the first, Atx binds to yPDI between domains b and b’ (bb’-model or binding site), while according to the second, it binds at domains a’ and c of yPDI (a’c-model or binding site). Both solutions are displayed on the same molecule of yPDI using the PyMOL program in two views rotated by 180^o^. Peptides cross-linked at Atx—yPDI interaction site mapping experiment are indicated by the same colours as in (B). Arrows point towards the active side of yPDI. Experimental details are in Materials and Methods.

To localize Atx binding sites on yPDI more precisely the interaction area between Atx and yPDI was mapped. To this end, the covalent complex between sulfo-SBED-Atx and yPDI was prepared and fragmented with α-chymotrypsin. The biotin-containing peptides were purified by avidin-affinity chromatography and RP-HPLC (Fig. 4A). The most abundant peptides (peaks 14, 16, 19, 22, 26, 28, 31 and 33 in Fig. 4A) were N-terminally sequenced, enabling their position in the primary structure of Atx or yPDI to be determined (Fig. 4B) and the structural elements in the two proteins that were cross-linked (colour coded in Fig. 4) to be defined. The biotinylated peptides were identified as elements of Atx *β*-sheet, *α*E-helix and the C-terminal unstructured area. In the case of yPDI such peptides originated from the bb`- and a`c-region. Identification of peptides clustered in two separate regions of PDI additionally supports the conclusion that yPDI contains two sites to which Atx can bind.

### Construction of a 3D model of the complex between Atx and yPDI

The structures of Atx and yPDI were docked as described in Materials and Methods, and by considering the structural constraints of the interaction revealed during the binding site mapping experiment. Two alternative docking procedures were employed. In the first, rigid-body docking protocol Hex 5.1 was used. Out of 89 solutions only 2 with the lowest calculated non-bonded energy suited also all of the established structural restraints. Both models were further refined by mild energy minimization allowing only smaller movements of side chain atoms to eliminate steric clashes produced by rigid-body docking. The definition of two final models was confirmed by the high ambiguity-driven docking (HADDOCK) method. Comparison of the two models resulted in only 1.1 Å average backbone Root Mean Square Deviation (RMSD) and, more importantly, in only 0.6 Å average backbone RMSD for the Atx—yPDI interaction surface. In the first model Atx binds to the hydrophobic and acidic surface between domains b and b’ of the yPDI (bb’-model). In the second, it binds to the acidic surface at the edge of domain a’ of the yPDI that extends to the neighbouring α-helix c (a’c-model) (Fig. 4C). Modelling thus confirmed the view stemming from affinity labelling and interaction area mapping experiments that Atx can bind at two diverse sites on yPDI.

**Fig 5:**
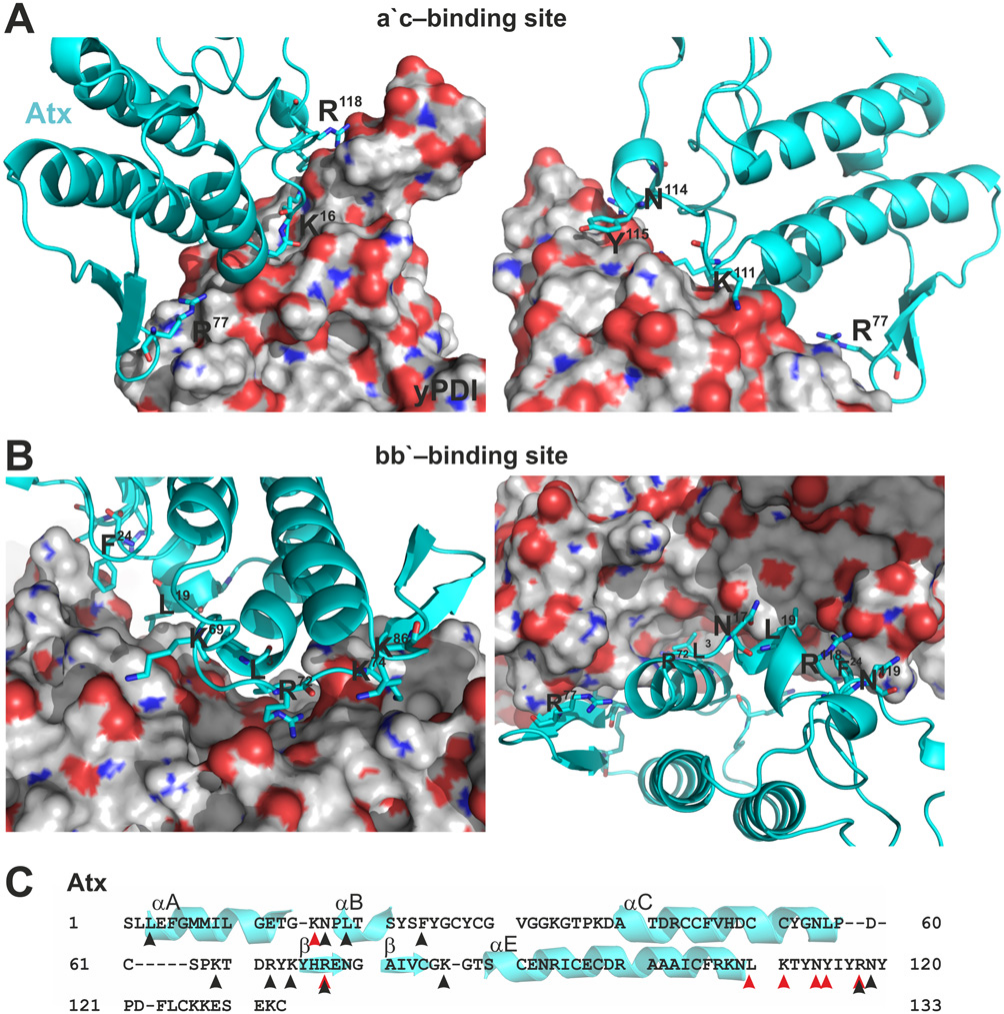
Detailed view of the Atx—yPDI interaction areas. (A) Details of the interaction between Atx and the a’c-binding site of yPDI. (B) Details of the interaction between Atx and the bb’-binding site of yPDI. Structures are prepared using the PyMOL program. yPDI is presented in space-filling mode while Atx in the ribbon mode, with side chains of the yPDI-interacting amino acids shown. (C) The yPDI-contacting amino acid residues of Atx are marked on its primary structure (arrowheads). Black arrowheads point at residues contacting yPDI at the bb’-binding site, while red at those contacting yPDI at the a’c-binding site. Elements of the secondary structure of Atx, α-helices and β-sheets, are shown behind corresponding parts of the primary structure.

The details of the interaction between Atx and yPDI at the two binding sites are shown in Fig. 5. The Atx-interacting surface on yPDI in the bb’-model is more extensive than that in the a’c-model. In the latter the electrostatic forces (ionic interactions and hydrogen bonds) between Atx and yPDI are predominant, while in the bb’-model the interaction between Atx and yPDI is supported also by hydrophobic interactions. For this reason it appears that the bb’-binding site is the primary binding site of sPLA_2_ on yPDI. The basic R77 and R118 are important amino acid residues in Atx that are involved in binding to the two binding sites on yPDI. In the bb’-model Atx also contacts yPDI with hydrophobic residues, L3, L19 and F24, two polar residues, N17 and N119, and four basic residues, K69, R72, K74 and K86. In the a’c-model Atx interacts with yPDI by two hydrophobic residues, L110 and Y115, a polar N114 and two positively charged residues, K16 and K111. Atx faces the bb’-binding site on yPDI with its interfacial binding surface (IBS). Atx binds, with a part of its IBS, to the a’c-binding site on yPDI [49]. Such a mode of binding to PDI should obstruct the binding of Atx to phospholipid membranes, inhibiting its phospholipase activity. However, no influence on the enzymatic activity of Atx has been detected in the presence of PDI. This apparent inconsistency can be explained by the almost 10.000-fold higher affinity of the sPLA_2_ molecule for PG membranes than to yPDI [16,50].

### Construction of a 3D model of the complex between Atx and hPDI

Human PDIs are heterogeneous with respect to their domain composition. While there are several hPDIs that contain the bb’ domain combination, none of them possesses the combination of a’c regions [17]. Our assumption that the bb’-binding site on yPDI is the main sPLA_2_-binding site is thus supported from the evolutionary point of view. The bb’-Atx interacting site on yPDI is highly homologous to corresponding regions in the bb’-hPDIs. Misfolded proteins, to be transported back to the cytosol, bind to this region in hPDI [26] as well as cholera toxin [51–53].

**Fig 6:**
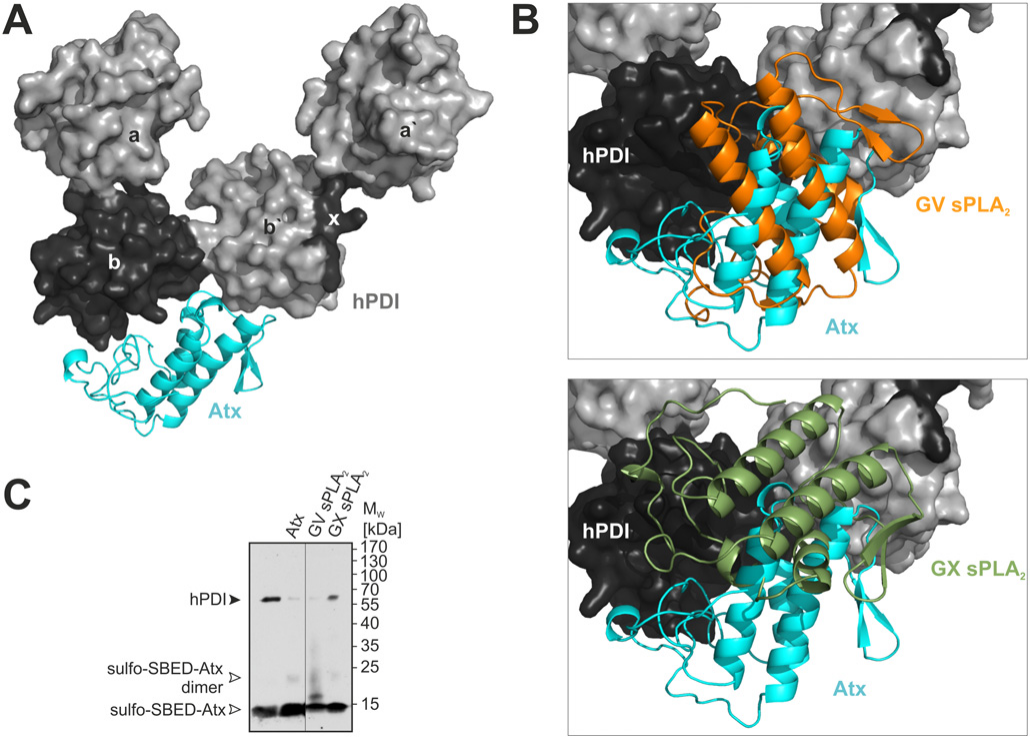
3D models of complexes between sPLA2s and hPDI. (A) Structure of hPDI was superimposed on the structure of yPDI in the complex with Atx bound to the bb'-binding site and the 3D model optimised by HADDOCK. The model is presented using the PyMOL program with hPDI in space-filling style and Atx in the ribbon style. (B) By substitution of Atx in the Atx—hPDI model with GIB, GIIA, GV or GX sPLA_2_, and HADDOCK molecular docking, the models of mammalian sPLA_2_s bound to hPDI were generated. GV sPLA_2_ (as also GIB and GIIA) occupies the same area on hPDI as Atx (upper picture), while GX sPLA_2_ occupies only part of this area (lower picture). (C) Heterologous competition of sulfo-SBED-Atx binding to hPDI by GV and GX sPLA_2_s confirms validity of the proposed sPLA_2_–hPDI 3D model. Human PDI was incubated with sulfo-SBED-Atx in the dark in the presence of 26 to 100-fold molar excess of AtxC, hGV or hGX sPLA_2_ over the sulfo-SBED-Atx. The photo cross-linking reaction was triggered by irradiation at 312 nm. SDS-PAGE analysis under reducing conditions was followed by Western blotting of the samples and biotin-specific detection on the nitrocellulose membranes. Black arrowhead is pointing at the biotinylated hPDI. White arrows are pointing at positions with biotinylated monomer and dimer of Atx. Experimental details are described in Materials and Methods section.

The first step in constructing the Atx-hPDI model was to superimpose the 3D structure of hPDI on the structure of yPDI in its bb’-model of the complex with Atx. The HADDOCK procedure was then implemented to obtain the energetically most favourable model of the complex (Fig. 6A). The docking resulted in a structure in which the position of the Atx molecule in the complex is substantially different from that in the complex with yPDI (Fig. 4C). Atx in the complex with hPDI is rotated by 180° and tilted by 5° with respect to its position in the complex with yPDI. Nevertheless, Atx interacts with hPDI *via* its IBS as in the case of yPDI. Interestingly, the same amino acid residues of the sPLA_2_, except R77, are involved in ionic, hydrophobic and hydrogen bond interactions with hPDI as with yPDI.

### Complexes between PDI and Atx-like mammalian sPLA_2_s

Toxins often take advantage of their similarity with endogenous molecules to conquer cells. Several mammalian sPLA_2_ molecules, for example GIB, GIIA, GV and GX sPLA_2_, are structurally highly homologous to Atx. GIB, GIIA and GV sPLA_2_ have been detected inside cells [9–11,54,55] while GX sPLA_2_ was not. The pathway of the sPLA_2_ cellular internalization has not yet been established. In order to consider the possible involvement of PDI in the cell trafficking of mammalian sPLA_2_s, we inspected *in silico* whether the latter are able to form complexes with PDI or not. The 3D structure of each of these molecules was superimposed on the structure of Atx in its complex with PDI. HADDOCK was then used to generate the energetically most favourable models. Besides some differences in relative orientation between respective sPLA_2_s in complexes, mammalian sPLA_2_s and Atx form very similar complexes with yPDI and hPDI. This is reflected in the average backbone RMSD values calculated for Atx and for each of the replacing sPLA_2_s in the complex with PDI. These values for GIB, GIIA, GV or GX sPLA_2_ in the case of the yPDI a`c-complex are 4, 3, 3 and 6 Å, respectively. For the yPDI bb'-complex the average backbone RMSD values for GIB, GIIA, GV and GX sPLA_2_ are much lower at 0.5, 2, 3 and 1 Å, exposing again the bb’-binding site on the yPDI as the main binding site for sPLA_2_s. Average RMSD values for comparison of the backbone atoms of Atx with those of GIB, GIIA, GV and GX sPLA_2_ in complex with hPDI are 3, 7, 4 and 12 Å. Consistent with the average RMSD values, GIB, GIIA and GV sPLA_2_ form complexes with hPDI similar to the Atx—hPDI complex (Fig. 6B, upper picture), while the interaction area of GX sPLA_2_ on hPDI is quite different (Fig. 6B, lower picture), only partially overlapping with those of Atx, GIB, GIIA or GV sPLA_2_.

To validate our strategy of modelling the complex between an sPLA_2_ and hPDI, we tested experimentally the ability of GV sPLA_2_, representative of mammalian sPLA_2_s that bind hPDI in the same way as Atx, and GX sPLA_2_, the mammalian sPLA_2_s that does not bind hPDI in the same way as Atx, to inhibit the binding of sulfo-SBED-Atx on hPDI. From the model we expected that GV sPLA_2_ should block biotin-tagging of hPDI more effectively than GX sPLA_2_. The results of experimental heterologous competition of sulfo-SBED-Atx binding to hPDI by GV or GX sPLA_2_ confirmed our prediction (Fig. 6C). GV sPLA_2_ indeed inhibited biotinylation of the hPDI more efficiently than the GX sPLA_2_. Our modelling approach thus gives relevant models of complexes between mammalian sPLA_2_s and hPDI. Models of the complex between Atx and hPDI, and the Atx-related mammalian sPLA_2_s and hPDI thus provide us with a structural understanding of how these proteins associate. This offers the possibility of facilitating the study of the role of PDI in the sPLA_2_ cell internalization process by targeted mutagenesis.

## Conclusions

The rat PC12 cell line has been shown to be a suitable neuron-like cell model to study the mechanism of the internalization of Atx, the snake venom neurotoxic GIIA sPLA_2_. Atx does not affect the morphology of these cells but is able to enter their cytosol. We showed that Atx and PDI substantially co-localize in PC12 cells, and, most importantly, are able to form a complex inside living PC12 cells. The results presented here strongly support the proposal that PDI, an oxido-reductase from the lumen of ER, is involved in the retrograde cell transport of Atx. Further study of the role of PDI in cell trafficking of Atx and structurally related mammalian sPLA_2_s included construction of a 3D model of the complex between Atx and hPDI. Based on this model, the complexes between GIB, GIIA, GV or GX sPLA_2_, and hPDI were described. These models predict that sPLA_2_s interact with hPDI in the region including its domains b and b’, the same region as that where unfolded proteins and cholera toxin bind on their retrograde pathway to the cytosol. The interaction area with hPDI is the same in the cases of Atx and the mammalian GIB, GIIA and GV sPLA_2_s, which have been detected intracellularly, while that of GX sPLA_2_, which has not so far been observed inside cells, is quite diverse in its location. In heterologous competition of Atx binding to hPDI, GV sPLA_2_ proved to be a better inhibitor than GX sPLA_2_. The validity of the sPLA_2_–hPDI 3D model was thus verified experimentally. It qualifies as an important tool to accelerate further insight into the role of PDI in assisting the retrograde transport of certain snake venom and mammalian sPLA_2_s from the cell surface and into (patho)physiological processes related to the intracellular action of these molecules.

## Supporting Information

S1 Fig The viability of PC12 cells is increased in the presence of Atx. (A) Non-differentiated (ND) and (B) NGF-differentiated (NGFD) PC12 cells were incubated with 100 nM AtxA, in its absence (control) or with 1% (w/v) Triton X-100 for the indicated periods of time. The viability of cells, determined using the MTS viability test, was calculated from A_490_ corrected for background absorbance and is presented relative to the control cells. Experimental details are described in Materials and Methods section.
(TIF)
