# Supplementary figures and images for: On the Role of Protein Disulfide Isomerase in the Retrograde Cell Transport of Secreted Phospholipases A2
Source: PLoS One. 2015 Mar 12;10(3):e0120692. doi: 10.1371/journal.pone.0120692 (PMC4357439; doi:10.1371/journal.pone.0120692)

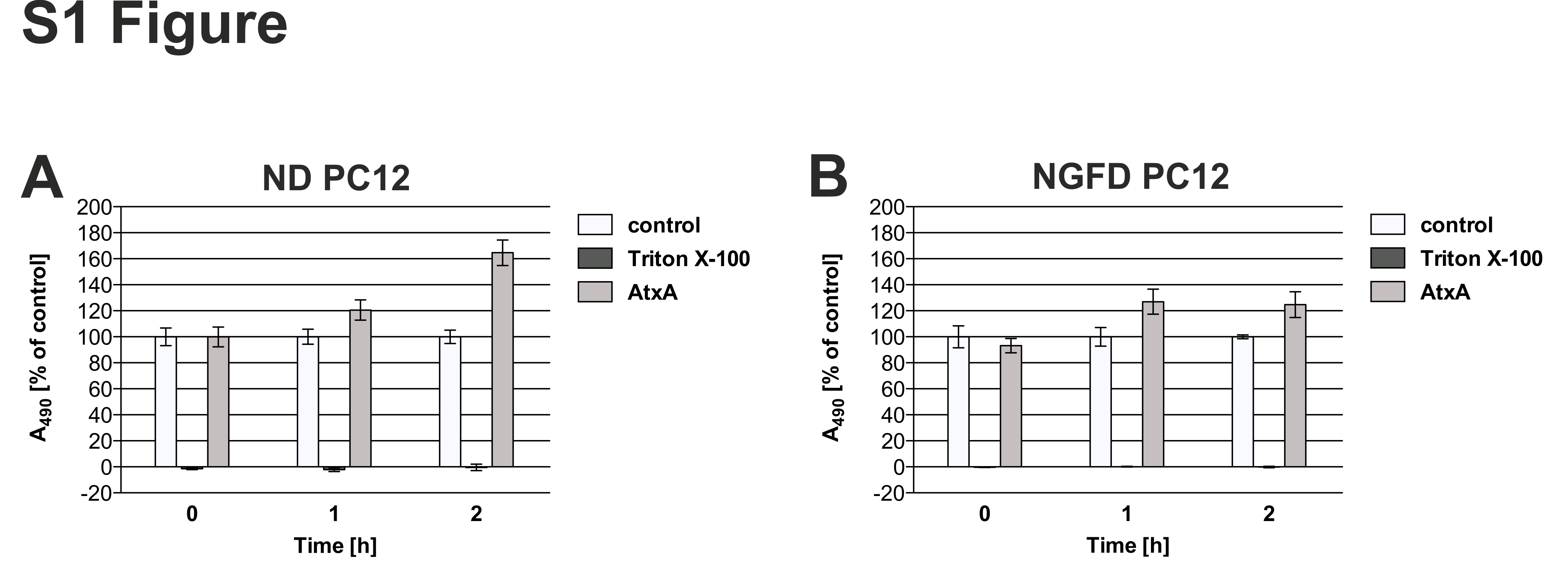

Supplement: S1 Fig — (A) Non-differentiated (ND) and (B) NGF-differentiated (NGFD) PC12 cells were incubated with 100 nM AtxA, in its absence (control) or with 1% (w/v) Triton X-100 for the indicated periods of time. The viability of cells, determined using the MTS viability test, was calculated from A490 corrected for background absorbance and is presented relative to the control cells. Experimental details are described in Materials and Methods section. (TIF) [file pone.0120692.s001.tif]
